# Selected Ion Extraction of Peptides with Heavy Isotopes and Hydrogen Loss Reduces the Type II Error in Plasma Proteomics

**DOI:** 10.1021/acsomega.4c05624

**Published:** 2025-01-02

**Authors:** Jaimie Dufresne, Zhuo Zhen Chen, Pallvi Sehajpal, Peter Bowden, Ja-An Ho, Cheng-Chih Richard Hsu, John G. Marshall

**Affiliations:** † Department of Chemistry and Biology, Faculty of Science, Toronto Metropolitan University, 350 Victoria Street, Toronto, Ontario M5B 2K3, Canada; ‡ Department of Chemistry, National Taiwan University, Taipei 10617, Taiwan

## Abstract

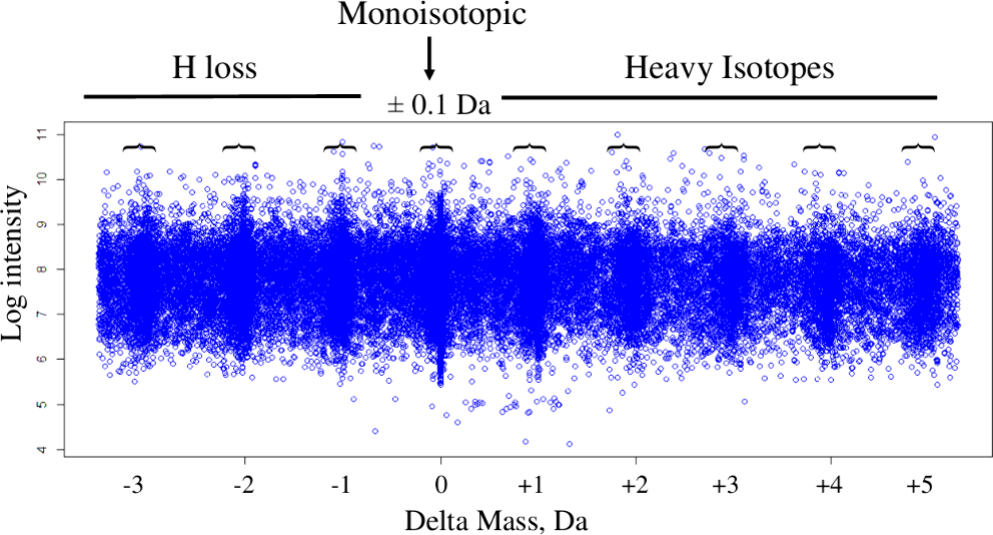

Naturally occurring peptides display a wide mass distribution after ionization due to the presence of heavy isotopes of C, H, N, O, and S and hydrogen loss. There is a crucial need for sensitive methods that collect as much information as possible about all plasma peptide forms. Statistical analysis of the delta mass distribution of peptide precursors from MS/MS spectra that were matched to 63,077 peptide sequences by X!TANDEM revealed Gaussian peaks representing heavy isotopes and hydrogen loss at integer delta mass values of −3, −2, −1, 0, +1, +2, +3, +4, and +5 Da. Human plasma samples were precipitated in acetonitrile, and the resulting proteins were collected over a quaternary amine resin, eluted with NaCl, digested with trypsin, and analyzed by nano liquid chromatography–electrospray ionization–tandem mass spectrometry (LC-ESI-MS/MS) with an orbital ion trap (OIT). Fragment spectra (MS/MS) generated from the OIT data were fit to human fully tryptic peptides by X!TANDEM, which led to the identification of 3,888 protein gene symbols represented by three or more peptides (*n* ≥ 3). The peptide counts to plasma proteins from experimental MS/MS spectra were corrected against 29 blank LC-ESI-MS/MS spectra and 30 million random MS/MS control spectra to yield 2,784 true positive proteins (*n* ≥ 3; *q* ≤ 0.01). Peptides identified by fragmenting ions with Gaussian heavy isotopes and hydrogen loss that were matched to known plasma proteins, such as albumin (ALB), were shown to be true positives and agreed with the peptide sequences identified in the monoisotopic peak. Accepting the ions from the monoisotopic peak alone (±0.1 Da) yielded only 382 plasma proteins (*n* ≥ 3; type I error *q* ≤ 0.01; type II error ∼86%). In contrast, accepting all ions within ±0.1 Da around the hydrogen loss, monoisotopic, and heavy isotopic peaks led to the identification of 963 proteins (*n* ≥ 3; *q* ≤ 0.01; type II error ∼60%). Using the power of the OIT to resolve the Gaussian peaks from heavy isotopes and hydrogen loss resulted in the identification of three times more proteins with high confidence and a much lower type II error than analyzing peptides from the monoisotopic peak alone. The resolving power of the OIT may be exploited to increase observation frequencies and provide greater proteomic coverage and statistical power in comparative proteomics studies.

## Introduction

Selective ion extraction is a well-established method to obtain specific information from mass spectra.^1^ Naturally occurring peptides display a wide distribution of masses after ionization due to the incorporation of heavy isotopes of C, H, N, O, and S and hydrogen loss.^2−6^ There is a crucial need for the sensitive collection of as much information as possible from all peptide forms. Simple plasma chromatography methods led to the detection of only a few hundred proteins, and so 2- or 3-dimensional chromatography techniques were developed to improve sensitivity.^7−10^ Here, analysis of the delta mass distribution of plasma peptides identified by X!TANDEM revealed the presence of Gaussian peaks from hydrogen loss or rearrangement with delta mass values of −3, −2, and −1 Da, a monoisotopic peak at 0 Da, and heavy isotope peaks at +1, +2, +3, +4, or +5 Da as expected.^3−6^ One solution to the challenge presented by a low monoisotopic signal has been to grow cells in the presence of light isotopes only so that all signals would be found in the monoisotopic mass.^3^ However, because plasma contains heavy isotopes, the challenge to obtain sensitivity can be addressed by including signals from Gaussian heavy isotope peaks and hydrogen loss. Here, the high resolution of the orbital trap instrument was exploited to select precursor ions within the Gaussian peaks from monoisotopic peptides as well as those with heavy isotopes and hydrogen loss. Tryptic peptides from plasma proteins identified by MS/MS spectra via X!TANDEM displayed delta mass values ranging from −3.0 to +5.0 Da that show low type I (false positive) error estimated using two independent methods, i.e., protein gene symbol *p*-values and false discovery rate (FDR) corrected *q*-values but also with a Monte Carlo simulation against random MS/MS spectra as the control.^11^ The type I error rate can be measured directly from the *p*-value from the exact goodness of fit of MS/MS spectra in X!TANDEM^12^ and can be corrected to an FDR *q*-value using the well-established method of Benjamini and Hochberg.^13^ The validity of protein *p*-values and FDR *q*-values determined by X!TANDEM^12^ for standards, complex eukaryotic cells, and plasma samples has been previously confirmed by the Chi-square (χ^2^) test using noise and random MS/MS spectra as controls.^14−16^ Experiments with unit-resolution ion traps provided strong evidence indicating that identification of peptides from the fit of MS/MS spectra (±0.5 Da) alone was accompanied by a low error rate.^12,17−22^ A Monte Carlo comparison^23−26^ of observation frequencies from an authentic protein library versus a random amino acid library revealed that the type I error for proteins observed from three or more peptides was low.^22^ Furthermore, analyses of purified proteins or protein samples with noise and random MS/MS spectra as controls^14,15,22,27−29^ revealed that the level of confidence increased with replicate observations.^30^ Identification of synthetic peptide standards from MS/MS spectra by high-resolution MS provided powerful evidence for the validity of peptide identification based on MS/MS fragmentation alone.^31^

Previous studies have shown that high-resolution orbital traps were in excellent agreement with the results generated from the more sensitive low-resolution linear quadrupole ion trap (LTQ).^32^ Identification of the purified standards by the LTQ was optimal at delta mass settings of ±3 Da around the monoisotopic mass.^14,15^ Although the LC-ESI-MS/MS method for identifying peptides works well in many tissues and cell types, it has been challenging to apply to plasma samples. Many of the proteins in plasma are detected at a low frequency, leading to results with poor statistical power for disease diagnosis or drug treatment comparisons. A previous study has shown that plasma proteins could be separated by micropartition chromatography, digested with trypsin, and identified and enumerated by microflow electrospray with a Paul ion trap to yield 4,396 distinct proteins from fully tryptic peptides by the rigorous X!TANDEM algorithm.^33^ Findings from the simple ion trap were confirmed independently by members of the Human Proteome Organization, which identified 4,395 proteins from human plasma.^34^ Statistical comparisons of the protein lists reported by different laboratories based on independent peptide sequences revealed strong agreement on more than 3,000 plasma proteins (all *n* ≥ 3) from MS/MS fragmentation.^35−37^ Similarly, a form of batch chromatography with a panel of nanomagnetic particles was used to identify ∼2,000 serum proteins using the sensitive SEQUEST algorithm.^38^

In this study, the preparation of human plasma with acetonitrile precipitation and quaternary amine (ammonium) (QA) chromatography for an orbital trap led to the identification of 2,784 distinct human protein gene symbols (*n* ≥ 3; *q* ≤ 0.01) from the MS/MS spectra of fully tryptic peptides alongside noise (blank injection) and random controls (30 million random MS/MS spectra).^39,40^ Under these controlled conditions, it was possible to estimate the type I and type II (false negative) error rates of the heavy isotopes and hydrogen loss peaks within a specified delta mass tolerance versus those associated with the monoisotopic peak alone. The process yielded low *p*-values and FDR *q*-values and good resolution from the observation frequencies of blank noise and random MS/MS spectra controls. The fit of peptides by MS/MS alone was combined with subsequent selected ion data extraction based on computed delta mass values to control type II error in plasma LC-ESI-MS/MS.

## Results

A total of 524,867 MS/MS spectra were collected from peptide precursors with 1000 or more arbitrary detector counts that were matched to peptides from a library of 157,636 human protein accessions, leading to the identification of 3,888 protein gene symbols (*n* ≥ 3) by the rigorous X!TANDEM algorithm.^12^ The observation frequencies determined from the experimental orbital trap results were corrected against those of the analytical control (29 blank LC-ESI-MS/MS runs; 493,541 MS/MS spectra) and the statistical control (30,000,000 random fragmentation spectra) to yield 63,077 peptides from 2,784 true-positive protein types (∼14.5% of proteins gene symbols; *n* ≥ 3, *q* ≤ 0.01) that were identified based on MS/MS spectra and where the type II error rate approached zero. Accepting the MS/MS results from the monoisotopic peak alone (delta mass of 0 ± 0.01 Da) led to the detection of only 302 known plasma proteins with low type I error rates. However, including MS/MS spectra from heavy isotopes and hydrogen loss (±0.1 Da) facilitated the identification of 963 plasma proteins (*n* ≥ 3; *q* ≤ 0.01) with a similar low type I error rate but a much lower type II error rate. Total experimental error was dramatically reduced when the resolving power of the orbital trap was used to generate MS/MS spectra from heavy isotopes, and hydrogen losses were observed by sharp Gaussian peaks.

### Workflow

Proteins from nine human plasma samples were precipitated in ACN, collected over a QA strong anion exchange resin, and then digested with trypsin.^39,40^ The tryptic peptides were analyzed by LC-ESI-MS/MS on the tribrid axially harmonic OIT, and the results were collected in an SQL Server database. The nonredundant Best Fit Per Spectra (BFPS) determined with a wide mass window (−3 to +5 Da) were mapped to single best fit per peptide sequence and charge state using an SQL Server that effectively controlled the potentially large type I error generated by the redundant use of MS/MS spectra.^11,14,15,41−43^ The orbital trap data displayed high mass resolution that separated heavy isotopes and hydrogen loss into nine mass precursor mass peaks at −3, −2, −1, 0, +1, +2, +3, +4, and +5 Da. The MS/MS spectra collected from all peptides were matched to true positive peptides using the X!TANDEM algorithm that served as the benchmark to estimate type II error after correction against noise and random MS/MS controls (Supporting Information Figure 1).

### Peptide Distributions

**Figure 1:**
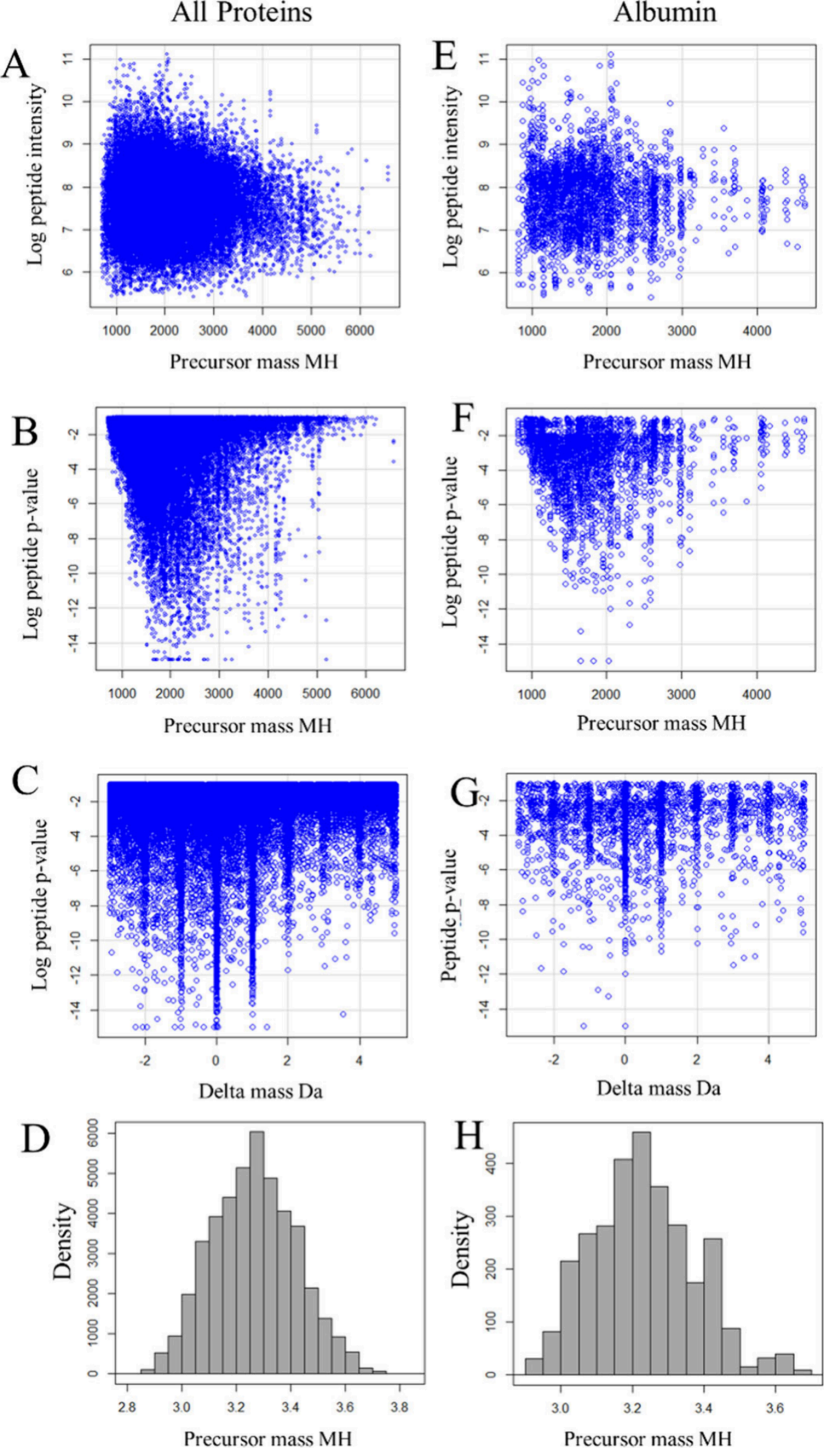
Scatter plots and distributions of the calculated peptide mass or delta mass values plotted against the log peptide intensity or log peptide *p*-value from all proteins (left) or true positive albumin (ALB) alone (right). A,E Scatter plot of precursor mass (MH) versus log intensity for all peptides; B,F, Scatter plot of MH versus log *p*-value for all peptides; C,G, Scatter plot of delta mass (Da) versus log *p*-value from all peptides; D,H, Histogram of the log MH versus density of all peptides.

The masses of the peptides derived from all proteins (Figure 1A) versus those from albumin (ALB, true positive) alone (Figure 1E) were focused from 1000 to 4000 Da with a maximum value around 6500 Da and with an average peptide mass of 1945.6 Da. The *p*-values of individual peptides were as low as *p* ≤ E–16 (i.e., machine zero), with most plasma proteins exhibiting *p*-values in a range similar to that determined for ALB (Figure 1B and 1F). A plot of the peptide *p*-values versus delta mass revealed highly significant MS/MS fits to peptide sequences detected at delta mass values of −3, −2, −1, 0, +1, +2, +3, +4, and as high as +5 Da around the monoisotopic peak (Figure 1C and 1G). As shown in the scatter plots, the peptide data form sharp Gaussian peaks from heavy isotopes and hydrogen loss at −3, −2, −1, 0, +1, +2, +3, +4, and +5 Da.^3−6^ Computations of the isotopic distribution of peptides from common plasma proteins that represent the range of peptide masses observed herein, including S100 calcium-binding protein A (S100A), alpha-2 HS-glycoprotein (AHSG), ALB, apolipoprotein A1 (APOA1), and complement component 6 (C6), revealed a significant number of heavy isotope peaks at the low end of the distribution (i.e., at or below ∼1000 Da). The signals obtained from peptides with heavy isotopes exceeded those from the monoisotopic mass by 1800 to 2000 Da; the signal from the monoisotopic peak alone represented only a small fraction of the signal obtained from the larger peptides (Supporting Information Figure 2). The log_10_ precursor mass (MH) distributions of all the X!TANDEM MS/MS correlations of plasma proteins yielded a Gaussian distribution from ∼500 to 6500 Da in which about half of the peptides were >1945 Da (Figure 1D and 1H). An analysis of the experimental data in SQL/R with a focus on delta mass units away from the monoisotopic peak explained most of the variation observed among the XTANDEM peptides (Supporting Information Figures 3, 4, and 5).

### Ion Extraction at Gaussian Hydrogen Loss and Isotopes

**Figure 2:**
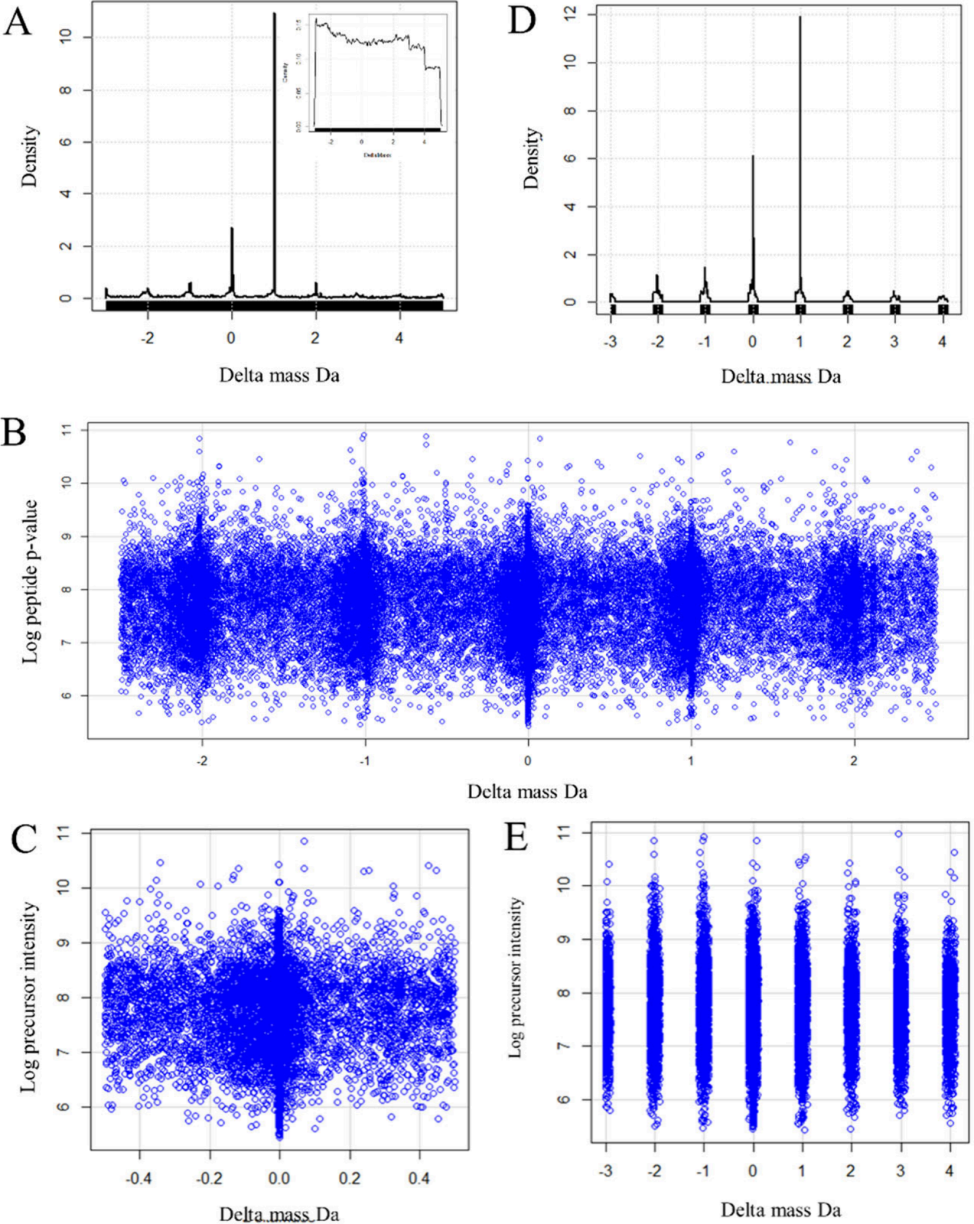
Density and scatter plots of peptide delta mass versus log_10_ precursor intensity for peptides from all plasma proteins. A, Density plot of the delta mass values (Da) from 67,898 MS/MS fit to plasma peptides; B, Scatter plot of peptide delta mass values (Da) versus the log peptide intensity values for the −2 to +2 Da precursor delta mass range for MS/MS fit to plasma peptides; C, Scatter plot of peptide delta mass values (Da) versus the log peptide intensity values for the −0.5 to +0.5 Da precursor delta mass range for MS/MS fit to plasma peptides; D, Density plot of the ion-extracted rearrangement and heavy isotope integer delta mass values (Da) ± 0.1 Da; E, Scatter plot of the delta mass values (Da) of the ions extracted from hydrogen loss and heavy isotope integer values ±0.1 Da.

Gaussian density analysis of the precursor delta mass data revealed a series of significant peaks representing the expected heavy isotopes and hydrogen loss at −3, −2, −1, 0, +1, +2, +3, +4, and +5 Da (Figure 2A). In contrast, the peptides from the 30 million random spectra were randomly distributed with respect to the delta mass (Figure 2A, inset). Scatter plots indicated that the heavy isotope and H-loss peaks were typically wider than ±0.1 Da (Figure 2B and 2C). Gaussian density analysis of the precursor delta mass values revealed that the +1 heavy isotope typically exceeded the density of the monoisotopic mass and that the signals from heavy isotopes and hydrogen loss were significant. It was possible to exploit the resolution and mass accuracy of the orbital trap to facilitate the selection of a narrow mass window around the H loss and heavy isotope peaks to increase sensitivity and reduce type II error with a specified mass tolerance value (e.g., ±0.1 Da) (Figure 2D,E).

### Estimating Type I Error from MS/MS Spectra Alone

**Figure 3:**
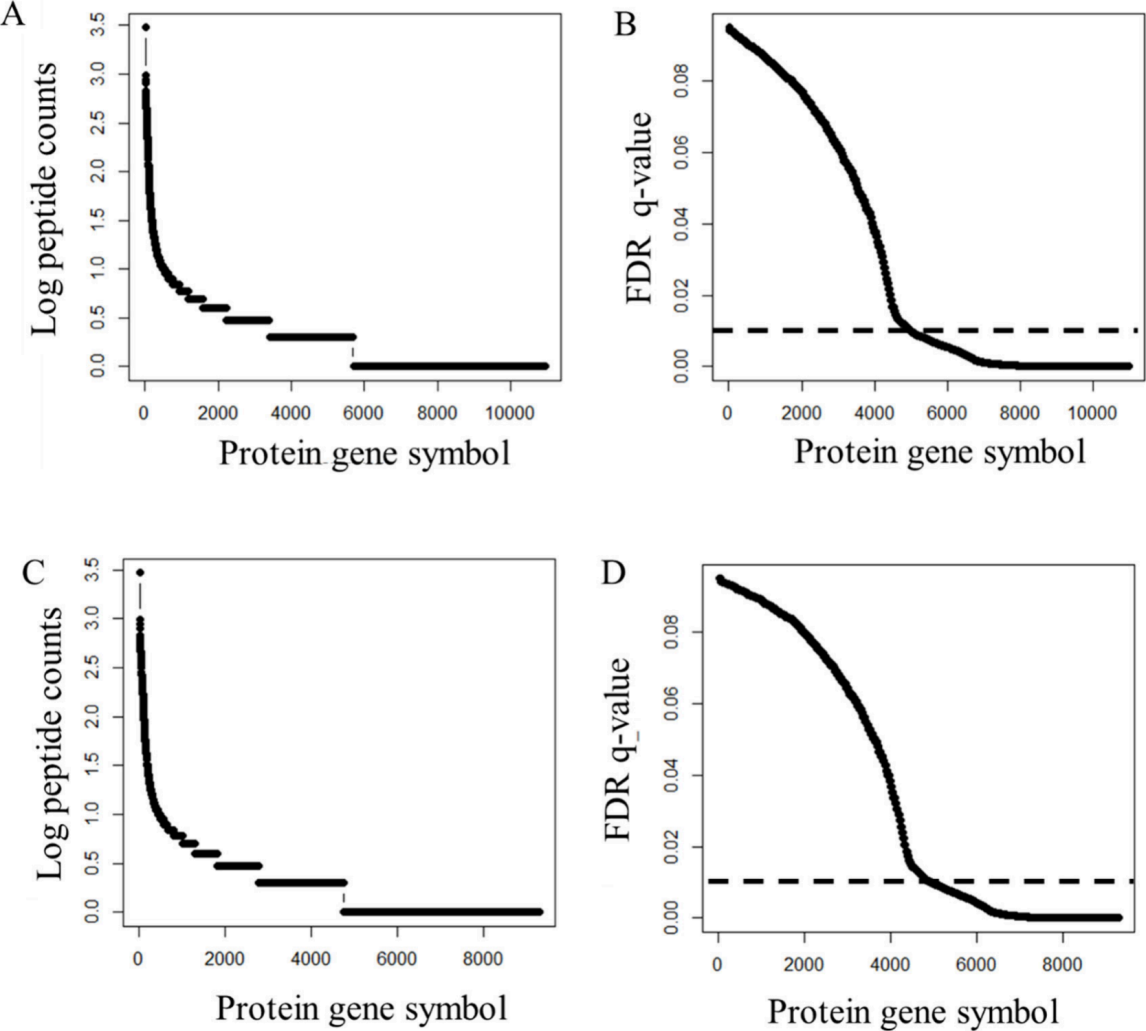
Estimating the type I error rate from peptide count from the fit of MS/MS spectra using the exact *p*-value, FDR corrected *q*-value, and Monte Carlo simulation with 30 million random MS/MS spectra. Panels: A, The peptide-to-protein observation frequencies (counts) of the OIT from the fit of all experimental MS/MS spectra corrected by subtracting the peptides observed in blank injection MS/MS spectra; B, Plasma proteins with FDR *q*-values ≤0.01 identified in all MS/MS spectra after subtraction of peptides from blank injection MS/MS spectra at the peptide level; C, Peptide-to-protein observation frequencies (counts) from the OIT and the fit of all experimental MS/MS spectra corrected by subtracting the peptides observed in blank injections and 30,000,000 random MS/MS spectra; D, Plasma proteins with FDR *q*-values ≤0.01 identified in all MS/MS spectra after subtraction of peptides from blank injections and 30,000,000 random MS/MS spectra and Monte Carlo simulations.

Analyzing plasma peptides by LC-ESI-MS/MS should result in peptides that correlate with known plasma proteins (Table 1).^35,36,44,45^ Here, experimental MS/MS spectra from nine plasma samples (25 μL) were matched to 3,888 protein gene symbols (*n* ≥ 3) by X!TANDEM (Figure 3A) with a low mean type I error rate of (*p* ≤ 0.01) (Figure 3B). After correcting the protein/gene symbol observation frequencies against those resulting from blank injections and random MS/MS spectra, the X!TANDEM algorithm generated a corrected list of 2,784 high confidence protein gene symbols with *n* ≥ 3 (Figure 3C) and FDR *q*-values ≤0.01 (Figure 3D) that prominently include the known true positive proteins of plasma (see Supporting Information).

**Table 1:** Effect of Ion Extraction of the Monoisotopic Peak (Delta Mass 0 ± Tolerance as Indicated) on the Number of Protein Gene Symbols Represented by Three or More Peptides (*n* ≥ 3) Together with the FDR (*q* or Type I Error), Number of Peptide Sequences (MS/MS), and Type II Error Rate^a^

Treatment	*n* ≥ 3	FDR	MS/MS	Type II error
Monoisotopic ±0.01	302	*q* ≤ 0.01	12381	89%
Monoisotopic ±0.05	351	*q* ≤ 0.01	14456	87%
Monoisotopic ±0.1	382	*q* ≤ 0.01	15619	86%
Monoisotopic ±0.2	423	*q* ≤ 0.01	16997	85%
Monoisotopic ±0.3	452	*q* ≤ 0.01	18023	84%
Monoisotopic ±0.4	483	*q* ≤ 0.01	19078	83%
Monoisotopic ±0.5	525	*q* ≤ 0.01	20169	81%
All MS/MS corrected	2,784	*q* ≤ 0.01	63777	0%
All MS/MS not corrected	3,888		71942	

a The precursor ions were fragmented by MS/MS and fit to peptides of 3,888 proteins by X!TANDEM. Peptides with similar observation frequencies in the blank noise recordings (493,541 MS/MS) or random MS/MS spectra (30,000,000 MS/MS) were removed to yield 2,784 true positives with a type II error rate approaching 0% (*n* ≥ 3; type I error ≤0.01). The type I error shown is computed from the *q*-values of the accepted proteins within the mass window indicated. Type II error was computed with respect to all of the peptides fit by MS/MS spectra by the X!TANDEM algorithm after correction by noise and random MS/MS spectra.

### Selection of the Monoisotopic Mass

Examining the effect of monoisotopic selective extraction width on the apparent false negative error rate of the most abundant plasma proteins showed that in general relaxing mass tolerance increased the true positive peptides, as confirmed by the fit of the MS/MS spectra (Supporting Information Table 1). The corrected type I error rate (i.e., FDR) for the proteins identified in all monoisotopic ion extractions estimated from the X!TANDEM fitting of the MS/MS fragments was acceptable (*q* ≤ 0.01) for the proteins identified in all monoisotopic ion extractions (Table 1). In contrast, the type II error rate based on the delta mass values varied significantly between the extraction methods. The major proteins identified in this analysis included ALB, apolipoproteins, and several other extracellular plasma proteins (i.e., true positives) that have been identified in the literature.^44^ However, filtering the results around the monoisotopic mass alone rejected many peptides from ALB as well as those from other common plasma proteins, including macroglobulin, protease inhibitors, apolipoproteins, complement, fibrinogens, clotting factors, clusterin, hemopexin, haptoglobin, and many others, all true positives and among the expected results. Thus, disregarding these peptides effectively creates a form of false negative (type II) error. Here, restricting the mass window to ±0.01 Da around the monoisotopic peak led to the identification of 302 known plasma proteins (*n* ≥ 3), while 382 proteins were identified by extending the mass window around the monoisotopic mass to ±0.1 Da with a negligible increase in type I error rate.

### Selected Ion Extraction of Heavy Isotopes and Hydrogen Loss

The type I error rates from the fit MS/MS spectra generated by X!TANDEM were uniformly low for all the delta mass filter settings (*q* ≤ 0.01), but the type II error rate (false negative) varied sharply from 0% to 65% based on the delta mass tolerance of ±0.1, ±0.2, or ±0.5 Da. The peptides and proteins from heavy isotopes and hydrogen loss were frequently the same peptides and proteins identified from the monoisotopic peak (Supporting Information Table 2). Accepting the peptides detected within ±0.1 Da of the hydrogen loss and heavy isotope peaks resulted in 963 proteins with negligible type I error rates (*n* ≥ 3; *q* ≤ 0.01). Thus, accepting peptides within a tolerance around the hydrogen loss and isotope peaks sharply increased the number of proteins identified, with no concomitant increase in type I error (Table 2). Almost half of the true positive protein gene symbols may be identified from the monoisotopic together with the +1 Da isotope ±0.1 Da (471, *n* ≥ 3) compared to all integer mass value (−3, −2, −1, 0, +1, +2, +3, +4, +5 Da) ± 0.1 Da that show 963 protein gene symbols with three or more peptide observations (*n* ≥ 3).

**Table 2:** Effect of Ion Extraction of the Gaussian Peaks from Heavy Isotopes and Hydrogen Loss (−3, −2, −1, 0, +1, +2, +3, +4, +5 Da ± Mass Tolerance As indicated) on Plasma Peptide Counts (*n* ≥ 3), Average *q*-Values, and Type II Error^a^

Treatment	*n* ≥ 3	FDR	MS/MS	Type II error
Hydrogen and Isotopes ±0.1 Da	963	*q* ≤ 0.01	35897	65%
Hydrogen and Isotopes ±0.2 Da	1,547	*q* ≤ 0.01	44662	29%
Hydrogen and Isotopes ±0.3 Da	1,981	*q* ≤ 0.01	50742	14%
Hydrogen and Isotopes ±0.4 Da	2,388	*q* ≤ 0.01	57394	14%
Hydrogen and Isotopes ±0.5 Da	2,784	*q* ≤ 0.01	63777	0%
All MS/MS Corrected	2,784	*q* ≤ 0.01	63777	0%
All MS/MS Not Corrected	3,888		71942	

a The MS/MS fragmentation spectra were fit to peptides within plasma protein gene symbols using the X!TANDEM algorithm. Peptides with similar observation frequencies in the blank noise recordings (493,541 MS/MS) or random MS/MS spectra (30,000,000 MS/MS) were removed to yield 2,784 true positives with the type II error rate approaching 0% (*n* ≥ 3; type I error *q* ≤ 0.01; type II error ∼0). The type I error shown is computed from the *q*-values of the accepted proteins within the mass window indicated. Type II error was computed with respect to all the peptides fit by MS/MS spectra by the X!TANDEM algorithm after correction by noise and random MS/MS spectra.

### Effect of Selective Ion Extraction on True Positive Albumin (ALB)

**Figure 4:**
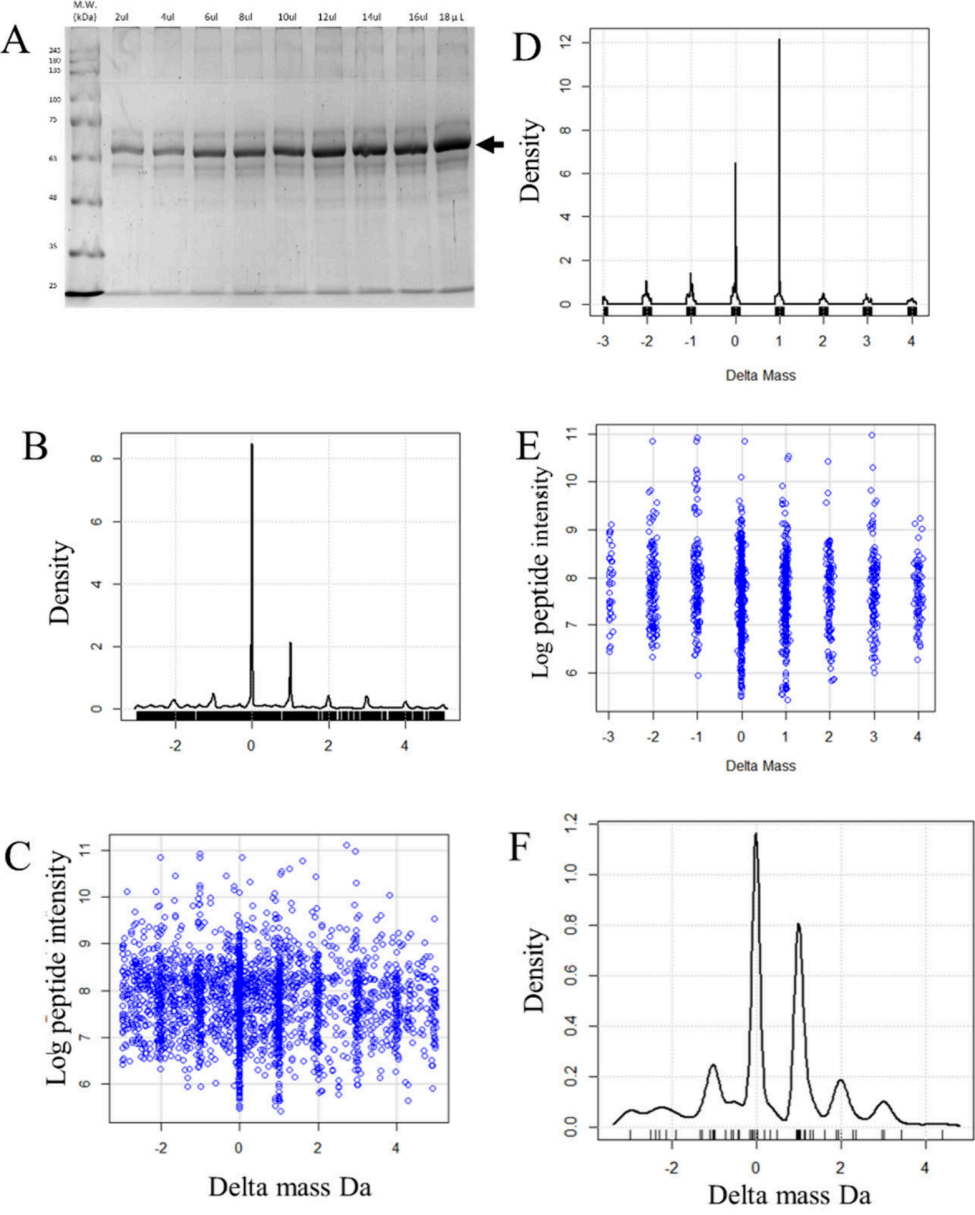
Analysis of plasma albumin (ALB; a known true positive) and its most common true-positive peptide, EQLKAVMDDFAAFVEK. A, Tris-glycine 12.5% SDS-PAGE of a human plasma sample in a serial dilution. Plasma was diluted 50 times in the SDS-PAGE sample buffer prior to loading the indicated volume for electrophoretic separation. The arrow indicates the electrophoretic mobility of ALB, which is the predominant band found in large excess over all other plasma proteins; B, Density plot of the delta mass values from peptides matched to ALB from the fit of MS/MS to plasma peptides by X!TANDEM; C, Scatter plot of peptide delta mass values versus log peptide intensity for the peptides matched to albumin by X!TANDEM; D, density plot of the ions extracted from the Gaussian peaks at heavy isotopes and hydrogen loss ±0.1 Da that matched peptides from ALB; E, Scatter plot of the delta mass values of the heavy isotopes and hydrogen loss ±0.1 Da from ALB after ion extraction; F, The peptide delta mass density plot of the ALB peptide EQLKAVMDDFAAFVEK (Table 3).

**Figure 5:**
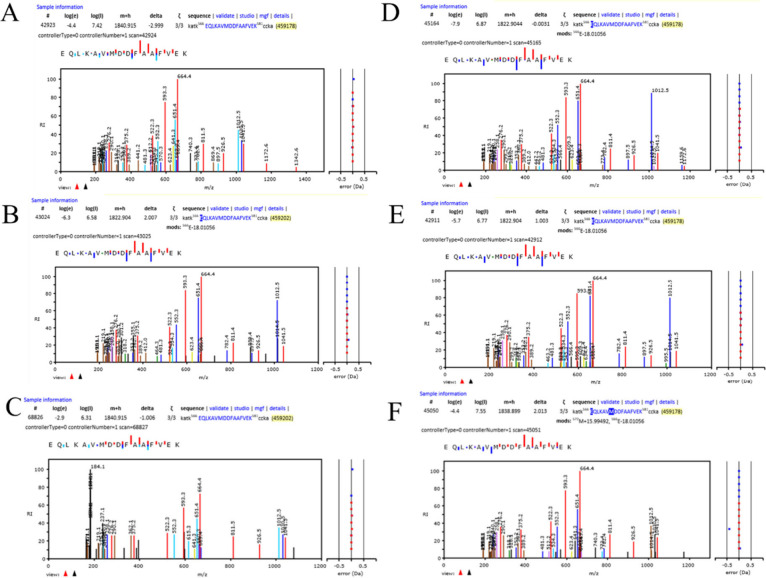
Tandem mass spectrometry analysis of the most common true-positive peptide, EQLKAVMDDFAAFVEK from plasma albumin. Similar MS/MS fragmentation spectra that match to the same peptide from albumin was observed from hydrogen loss and heavy isotopes peaks at delta mass values of −3, −2, −1, 0, +1, +2 Da. Panels: A, the −3 Da delta mass peptide MS/MS spectra; B, the −2 Da delta mass peptide MS/MS spectra; C, the −1 Da delta mass peptide MS/MS spectra; D, the 0 Da delta mass peptide MS/MS spectra; E, the +1 Da delta mass peptide MS/MS spectra; and F, the +2 Da delta mass peptide MS/MS spectra. The output of the X!TANDEM algorithm is shown without any alteration.

ALB is the major plasma protein, and so peptide matches to ALB may be considered true positive after subtracting blanks and correcting against random MS/MS spectra (Figure 4A). Selecting ALB peptides with precursors ±0.1 Da from the ALB hydrogen loss and heavy isotope peaks yielded higher observation frequencies and a sharp reduction in type II error while maintaining a low type I error rate (*p* ≤ 0.01) (Figure 4B, 4C, 4D, and 4E). The most common ALB peptide, EQLKAVMDDFAAFVEK, showed Gaussian peaks from −1 to +3 Da delta mass values (Figure 4F). The same peptide sequences observed near 0 delta mass were also detected in the hydrogen loss and heavy isotope peaks. Analysis of the LC-ESI-MS/MS peaks that included ±0.5 Da from the heavy isotope integer and H loss values within ±0.1 Da resulted in 84 identifications of the EQLKAVMDDFAAFVEK peptide (Table 3). Accepting the precursor masses (MHs) within ±0.1 Da from the monoisotopic peak yielded only 21 identifications of this common ALB peptide (i.e., a 75% type II error rate) compared to those from all the heavy isotope integers, and H-loss values resulted in 47 identifications (i.e., a type II error rate of 44%). It remains possible that some of the apparent heavy isotopes observed could result from peptides with multiple glutamine (Q) or asparagine (N); however, as Figure 5 shows the effect is observed in peptides that do not have multiple deamidation sites such as the peptide QLKAVMDDFAAFVEK from albumin that contains one glutamine (Q) and no asparagine (N), and so it is not possible that the increase in 2 to 3 Da results from the deamidation reaction of these amino acids. The most common ALB peptide was detected 26 times in the hydrogen loss and heavy isotope peaks, which must be considered true positive. Thus, including the hydrogen loss and heavy isotope peaks reduced the total experimental error (Supporting Information Table 1). The orbital trap mass spectrometer measures peptide mass to much less than a Dalton, but the peptides observed from MS/MS spectra showed peaks at exactly −1, −2, and −3 Da. Since there are few other modifications that result in the loss of 1 Da, the data clearly indicates that there has been a net loss of up to three hydrogens (Table 3 and Figure 5).

**Table 3:** Effect of Ion Extraction Treatments on the Identification and Error Rate in Analysis of the Serum Albumin Peptide EQLKAVMDDFAAFVEK with a Tolerance of ±0.1 Da around the Monoisotopic Peak Alone and Peaks from Hydrogen Loss and/or Heavy Isotopes^a^

EQLKAVMDDFAAFVEK			Type I error	Type II error
Delta Mass Selected	mean *p*-value	*n*	Cumulative *p*-value	
Monoisotopic +3 Da ± 0.1	0.006275	4	1.55 × 10^–09^	
Monoisotopic +2 Da ± 0.1	0.001685	6	2.30 × 10^–17^	
Monoisotopic +1 Da ± 0.1	0.006194	9	1.34 × 10^–20^	
Monoisotopic 0 Da ± 0.1	0.001848	21	4.02 × 10^–58^	75%
Monoisotopic –1 Da ± 0.1	0.003692	6	2.53 × 10^–15^	
Monoisotopic –2 Da ± 0.1	0.000785	2	6.16 × 10^–07^	
Monoisotopic –3 Da ± 0.1	0.000038	1	0.000038	
All rearrangements and isotopes ±0.1 Da	0.005467	47	4.72 × 10^–107^	44%
All MS/MS	0.008809	84	2.37 × 10^–173^	0%

a The MS/MS fragmentation spectra were fit with the X!TANDEM algorithm to EQLKAVMDDFAAFVEK (Figure 5). Note that selecting only the ±0.1 Da window around the monoisotopic peak results in a low observation count with a large type II error and therefore a large total error.

### Comparison of MS/MS Fit to Monoisotopic Precursor Mass

**Figure 6:**
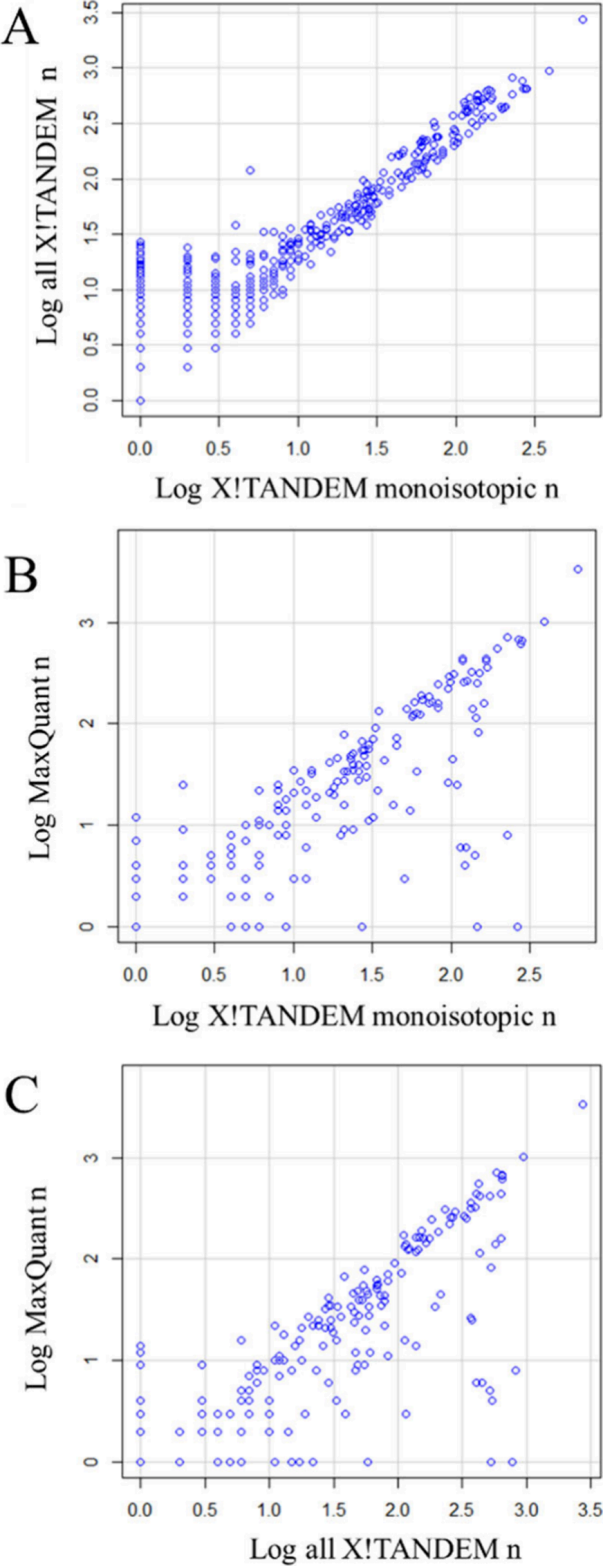
Agreement between the monoisotopic mass from X!TANDEM and MaxQuant versus all data including heavy isotopes, hydrogen loss, and other precursor mass values. Panels: A, regression of all corrected X!TANDEM observation counts (*n*) onto those of the monoisotopic precursor mass [Multiple R-squared: 0.8074, F-statistic: 5189 on 1 and 1238 DF, *p*-value: < 2.2e^–16^]; B, regression of all MaxQuant observation counts (*n*) onto all corrected X!TANDEM monoisotopic precursor mass observation counts [Multiple R-squared: 0.5358, F-statistic: 184.7 on 1 and 160 DF, *p*-value: < 2.2e^–16^]; C, regression of all MaxQuant observation counts (*n*) onto those of the X!TANDEM monoisotopic precursor mass [Residual standard error: 0.5248 on 191 degrees of freedom Multiple R-squared: 0.603, F-statistic: 290.1 on 1 and 191 DF, *p*-value: < 2.2e^–16^].

The X!TANDEM algorithm employs the classical statistical strategy of goodness of fit of the MS/MS spectra over a wide range of precursor masses to select the most significant match.^12^ The monoisotopic delta mass or any delta mass range can be selected from the X!TANDEM results in the SQL Server/R system. In contrast, the MaxQuant fits the monoisotopic mass but does not fit the heavy isotopes, hydrogen loss, or other masses.^46^ There was highly significant and quantitative agreement between all X!TANDEM results versus only those from the monoisotopic (±0.1 Da) mass (Figure 6A), between the monoisotopic results of MaxQuant versus all result from X!TANDEM (Figure 6B) and between the monoisotopic results of MaxQuant versus those of X!TANDEM (Figure 6C). The highly significant proportion between the monoisotopic mass observation counts, and those of the other mass values are a powerful statistical demonstration (*p* < 2.2e^–16^) that the peptides from the hydrogen and isotope peaks are in fact the same analyte as the monoisotope.

## Discussion

In this study, the resolving power of the OIT mass spectrometer was applied to human plasma proteomics to selectively extract ions with heavy isotopes and hydrogen loss within ±0.1 Da mass accuracy to gain sensitivity while controlling the type I error rate. Although many proteomic studies have focused on ways to reduce type I error,^22,28^ few have considered the effects of mass accuracy on type II error^14,15^ and thus total analytical error. A statistical analysis aims to obtain the lowest total error from the balance of false positive (type I) and false negative (type II) errors.^47,48^ Type I errors can be identified and addressed in subsequent biochemical experiments,^41,42^ but type II errors generate no hypotheses to test. Large type II errors are associated with considerable social cost because they result in failure to discover biomarkers, drugs, or drug targets that might be used for diagnostic and therapeutic purposes. Statistical methods, including the peptide counts,^22^ FDR *q*-values based on the fit of the experimental MS/MS spectra to the predicted peptide sequence,^12^ and Monte Carlo simulations of authentic versus random MS/MS spectra^28,29^ provided well-defined estimates of type I error rates.^16^ Estimates of type I error based on peptide counts,^22^*p*-values,^12^ and Monte Carlo simulations^28,29^ were all in agreement regarding a low type I error (*q* ≤ 0.01) for at least 2,784 protein gene symbols from the fit of MS/MS alone without regard to delta mass. Type I error is typically held to 5% (*p* ≤ 0.05) or 1% (*p* ≤ 0.01) in most statistical analyses to avoid incurring a large type II error.^48,49^ The statistical analysis presented here demonstrated that accepting only the monoisotopic peak at low delta mass led to a large type II error and, thus, a total error that was unacceptably large and showed poor sensitivity. In contrast, the fit of the MS/MS spectra showed good statistical power to discern true positive peptides. The simplest model that explains all data, contains few or no arbitrary elements, and that leads to testable predictions is that heavy isotopes and H-loss were recorded.

### Determination of Peptide Identity from MS/MS Spectra Alone

The X!TANDEM algorithm that directly fits MS/MS spectra (±0.5 Da) to peptides and generates a *p*-value via a rigorous but computationally intensive goodness of fit test that yields low rates of measured error when the data are corrected with analytical and statistical controls.^14,15,28,29^ In this study, where type I error could be established from the fit of MS/MS spectra alone by two independent classical statistical methods, goodness of fit, and Monte Carlo, it became possible to illustrate the deleterious impact of maintaining strict monoisotopic mass accuracy when attempting to achieve the full characterization of plasma proteins. In contrast to the limited computational capacity at the time at which proteomics studies were first initiated,^50−52^ 64-bit computations of goodness of fit and correction of each peptide MS/MS spectra from large proteomic data sets are now routinely feasible.^36^ Here, the fit of MS/MS spectra from a highly resolved orbital trap revealed large collections of peptides with heavy isotopes and hydrogen loss at the delta mass integer values of −3, −2, −1, +1, +2, +3, +4, and +5 that were correctly identified as the same peptides and proteins as those identified in the monoisotopic peak (i.e., delta mass near zero). Results from high-resolution MS agree with the isotope frequency predictions, notably, that as peptides exceed 1000 Da and approach 2000 Da, the density of the +1 Da isotope peak will exceed that of the monoisotopic peak.^53^ The inclusion of heavy isotopes and hydrogen loss revealed additional peptides ≥1945 Da, thereby increasing the observation frequency and coverage of the proteome. The chance that the same peptides were observed from the same proteins in the same proportions between the monoisotopic and other masses by random chance was about 2 e^–16^ and is powerful quantitative evidence that the peptides observed from MS/MS spectra alone agree with those of the monoisotopic mass and are therefore true positive.

### Exploiting Hydrogen Loss and Heavy Isotopes

The orbital trap is a powerful instrument for analyzing small molecules, particularly when tens of thousands of them are densely packed in the low mass ranges with relatively few isotopes and few fragments where mass accuracy is essential for identification. By sharp contrast, many or most peptides display heavy isotopes of C, H, N, O, and S or hydrogen loss, and so natural peptides ionize within a range of Gaussian peaks that can be up to 9 Da wide^3−6^ but show similar MS/MS spectra. Peptides are composed of amino acids of only 18 different masses that form fragmentation ladders; thus, the identity of many peptides can be directly assigned by the fit of MS/MS spectra alone with low type I error rates and without regard to precursor mass accuracy.^52,54^ Although *Escherichia coli* cells can be cultured *in vitro* with strictly monoisotopic elements, thereby avoiding a split between the monoisotopic signal and heavy isotope peaks,^3^ this approach would not work with medically and economically important human plasma samples. Moreover, culturing cells in light isotopes alone will not address the issue of hydrogen loss.^3−6^ Thus, for naturally occurring samples (e.g., human plasma), an alternative solution might be to include the Gaussian peaks around the heavy isotope delta and hydrogen loss delta mass values together to obtain improved observation frequencies for statistical analysis using MS/MS computations. The mass accuracy of the orbital trap can be utilized after the experiment has been completed to determine whether the peptides were derived from hydrogen loss or heavy isotopes. Computing the deviations from the hydrogen loss and heavy isotope peaks would thus exploit the hybrid instrument’s high resolution and its capacity to sample the Gaussian peaks, representing heavy isotopes and hydrogen loss to generate more observations and identifications of plasma proteins. The peptides identified within tolerance of the hydrogen loss and heavy isotope peaks were confirmed by agreement with the MS/MS spectra of peptides from the monoisotopic mass. Peptide bonds may show a double bond characteristic^55^ and hydrogen rearrangement that preceded fragmentation^4−6^ typically results in the *b* and *y* ion series from fragmentation at the peptide bonds with +1, +2, or +3 charge states that might correspond to the −1, −2, and −3 delta mass values observed here. Thus, the orbitrap and previous observations with electrospray ionization^5,6^ seem to reveal the presence of H-loss from metastable peptides in the gas phase at the precipice of *b* and *y* fragment ion formation. The simplest interpretation of the statistical data alone may be that peptide bonds take the characteristic of a double bond providing an H^+^ ion at high energy in the gas phase just prior to fragmentation producing the −1 Da, −2 Da, and −3 Da delta mass values corresponding to the *z* = +1, *z* = +2, and *z* = +3 charge state of peptides typically observed and may be important clues to the complex process of gas-phase peptide fragmentation into *b* and *y* ions.^56^

### Mass Accuracy and Error Rates

An analysis of the peptide delta mass values revealed that the Gaussian peaks from the +1 heavy isotope were resolved by the orbital trap^57^ with a density that may frequently exceed that of the monoisotopic peak.^2,3^ The MS/MS fragmentation of peptides with heavy isotopes and hydrogen loss may yield the same peptides and protein sequences with similar *p*-values and type I error observed from the monoisotopic mass. Restricting the search of the MS/MS spectra to the monoisotopic Gaussian peak resulted in a large type II error. Selecting all precursors with a defined mass tolerance around the integer representing the heavy isotope and hydrogen loss windows avoided substantial type II errors. Moreover, the isotopic distribution of peptides indicated that the monoisotopic peak may represent only a fraction of the signal detected for many peptides. Thus, selecting the monoisotopic peak alone may create a bias toward identifying smaller peptides from plasma proteins. Selecting precursor ions from a larger range surrounding each of the Gaussian peaks controlled the type II error rate and thus the total error and resulted in the identification and quantification of many more plasma proteins. A mass tolerance of ≥ ±0.1 Da around the hydrogen loss and heavy isotope peaks resulted in a reduction in the total experimental error, a finding that is in agreement with the scatter plot analysis of 63,077 delta mass plots overlaid together in SQL/R.

### Human Plasma Proteome

The data presented herein revealed that the fit of MS/MS spectra to specific human plasma peptides using the rigorous X!TANDEM algorithm with an OIT yielded 2,784 true positive proteins (*n* ≥ 3) after subtracting the analytical blank injection control and the 30 million random MS/MS spectra statistical control. The two independent statistical methods, i.e., computing a Monte Carlo simulation with random MS/MS spectra followed by computing the exact fit of peptides using X!TANDEM to generate protein FDR *q*-values, provided confidence in the resulting plasma proteome (*n* ≥ 3).^14,15,22,28,29,43^ Restricting the mass window to the monoisotopic peak alone led to much lower observation frequencies of the known plasma proteins, an apparent type II error that resulted in fewer peptides and proteins detected without substantial benefit, in terms of controlling the type I error rate. Utilizing the resolving power of the OIT to collect the MS/MS data from the Gaussian hydrogen loss and heavy isotope peaks resulted in higher peptide observation frequencies and the discovery of more human plasma proteins while controlling mass tolerance and total error. The MS/MS fitting algorithms have an advantage in terms of sensitivity and lower type II error from the selection of peptides that contain heavy isotopes or other mass modifications compared to algorithms that rely heavily on precursor monoisotopic mass.

## Conclusion

The study presented here revealed that a highly accurate trihybrid axially harmonic orbital trap mass spectrometer resolved the ion signals of peptides into Gaussian peaks around the monoisotopic mass and also around signals from peptides with hydrogen loss and heavy isotopes. The MS/MS spectra from the rearrangement and heavy isotope peaks correlated with the same peptides identified in the monoisotopic peaks. The simplest model that explains the presence of nine Gaussians spaced exactly 1 Da apart with the same peptide sequence observed at each 1 Da interval from the MS/MS spectra is that peptides with heavy isotopes and H-loss were fragmented. Accepting the peptides within extended windows around each Gaussian peak resulted in a large increase in observation frequencies and a sharp drop in type II error rates with no effect on type I error, thereby leading to a much larger, high-confidence plasma proteome. Thus, the resolving power of the orbital trap may be exploited to achieve a greater sensitivity without sacrificing type I error rates. Extracting precursor ions within a specific tolerance around peaks from heavy isotopes and hydrogen loss will thus increase the observation frequencies and provide greater proteomic coverage and statistical power in comparative proteomics studies, which is of great basic and clinical importance.

## Materials and Methods

### Materials

The Dionex UltiMate 3000 series Ultra-Performance Liquid Chromatography (UPLC), C18 Acclaim PepMap Nano column (75 μm ID, 25 cm length, C18), Fusion Lumos Orbital ion trap, and High-Performance Liquid Chromatography (HPLC)-grade water and acetonitrile (ACN) were obtained from Thermo Fisher Scientific (Waltham, MA, USA). The QA resin on the ceramic support was from BIORAD (Hercules, CA, USA). Sequencing grade trypsin was obtained from Promega (Madison, WI, USA). The normal human plasma was from St Michaels Hospital (Unity Health), Toronto, Canada REB# 20–078.

### Plasma Separation and Analysis

Nine human plasma samples (25 μL) were precipitated with 10 volumes of ACN and centrifuged at 15,000 relative centrifugal force (RCF) for 15 min at room temperature; the supernatant was then removed and the proteins were dried under a vacuum.^58^ The precipitated proteins were resuspended in 250 μL of 20 mM tris pH 8.85 loading buffer on ice with vortexing and centrifuged at 15,000 RCF for 5 min. Intact plasma proteins in loading buffer were collected over 100 μL of QA resin, washed with three column volumes (300 μL) of loading buffer, and eluted with two column volumes (200 μL) of 0.6 M NaCl in the same buffer. The refined plasma proteins were quantitated by Bradford assay^59^ against bovine serum albumin (BSA) standards prior to either SDS-PAGE analysis or directly digested in the liquid phase for LC-ESI-MS/MS.^60^ The proteins were separated by glycine sodium dodecyl sulfate-polyacrylamide gel electrophoresis (SDS-PAGE)^61^ followed by staining with Coomassie Brilliant Blue. Protein samples were digested with trypsin at a 1/100 ratio in 600 mM urea, 5% ACN in 20 mM tris pH 8.85 at 37 °C for 16 h.^60^ The digests were quenched with 5% acetic acid before the binding step on the ZipTip C18 resin.^33^

### Orbital Ion Trap (OIT)

The plasma peptides collected over a C18 ZipTip were eluted in 2 μL of 5% formic acid and 65% ACN and immediately diluted with 18 μL of 5% formic acid for injection via a 20 μL loop. The resulting peptides were analyzed over an Acclaim PepMap Nano LC column (C18, 2 μm particles with a pore size of 100 Å, ID: 0.075 mm × 250 mm) at 30 nL/min for 50 min at a gradient from 5 to 70% acetonitrile for the Thermo orbital ion trap Fusion Lumos (Q-Orbital ion trap-LTQ Tribrid MS) at the Department of Chemistry at National Taiwan University. The instrument was tested with the manufacturer’s calibration mixture. The electrospray was formed at 2000 V, and the ion transfer tube was maintained at 275 °C. The tryptic peptides were identified by MS scans from *m*/*z* 350 to *m*/*z* 1700 and followed by higher-energy collisional dissociation-MS/MS spectra of the most intense ions at a normalized collision energy of 32%. The MS survey scan was performed every 3 s (AGC target 5e5, maximum injection time of 50 ms), and all ions above the signal threshold were submitted for MS/MS (AGC target 5e^4^, maximum injection time of 50 ms) in order of decreasing intensity with previously selected ions dynamically excluded for 60 s. The isolation width was 1.4 Th.

### X!TANDEM Peptide MS/MS Spectra Correlation Analysis

A physical filter of at least 1,000 (E3) intensity counts for peptide parent ions was used to limit the type I errors.^14,15,28,29,32^ The fragmentation spectra were fit to the peptides of an annotated Uniprot library with 157,636 distinct human protein sequences using the X!TANDEM algorithm.^12^ The MS/MS spectra were fit to peptides with fully tryptic X!TANDEM settings.^12^ The X!TANDEM algorithm was set to search parameters of 2+ or 3+ precursor ions from 500 to 2000 *m*/*z* that correlated from −3.0 to +5.0 Da with fragments ±0.5 Da and a maximal accepted value of *p* ≤ 0.01.^14,15^ Tryptic cleavage rules ([RK]|[X] with up to 3 missed cleavages) were applied with potential modifications setting: modification of Cysteine (C) (+57.021464), potential modifications such as the oxidation of methionine (M) or tryptophan (W) (+15.994915), dioxidation of M or W (+31.98983), and deamidation of asparagine (N) or glutamine (Q) (+0.984016). The addition of a carboxy-terminal hydroxyl (+17.002735) or an amino-terminal proton (+1.007825) was also included with a maximum peptide *p*-value of *p* ≤ 0.01.

### Random and Noise MS/MS Controls

The sum of 29 blank (noise) injection LC-ESI-MS/MS experiments (493,541 MS/MS) served as the analytical control, and the observation frequencies of random MS/MS spectra (30,000,000 MS/MS) served as the statistical control. The observation frequencies of the experimental results were compared to a Monte Carlo simulation of random MS/MS spectra created with a modification of the random MS/MS spectra generator as previously described.^28,29^ The weighted average and range of precursor masses, the number of fragments, and the fragment range and mass distribution were set to match those of the experimental observations. The fragments were restricted to 150 to 2000 *m*/*z*. The experimental results were then corrected using the analytical control of blank injections with HPLC grade solvents over naïve columns^5,14,15^ and the statistical control of 30 million random MS/MS spectra.^28,29^

### Computational Analysis in Structured Query Language (SQL) and Statistical Analysis with R

The LC-ESI-MS/MS spectra, including the parent and fragment *m*/*z* and intensity values and the resulting peptide and protein identifications from X!TANDEM,^12^ were parsed into an SQL Server database.^36^ The inherent complex-key features of the SQL Server system permitted the MS/MS spectra to be evaluated based on a nonredundant BFPS data set. The proteins from the tryptic peptides were evaluated in an SQL Server with each MS/MS spectrum used only once and assigned to the best fit (if any) peptide. The observation frequencies of the peptide counts were corrected against the sum of 29 blank injection recordings (analytical control) and 30 million random MS/MS spectra (statistical control). Peptides from random MS/MS spectra typically accumulate in large or variable proteins with extensive sequences, for example, TTN, COL, HLA(MHC), AHNAK, MUC16, FAM230A (ncRNA), SYNE1, MACF1, and OBSCN, PCLO, DST, PLEC, SYNE2, NPR2, SPEG, Immunoglobulin (Ig) variants, IGFN1, and others. After selecting peptides well resolved from the analytical and statistical controls (χ2 ≥ 9), *p*-values^36^ and FDR-corrected *q*-values^13^ can be computed and the data distribution plotted using the R statistical system.^43^ Density plots were made with the default Gaussian settings in Rcmdr.

## Data Availability

The computed results are provided in the Supporting data. The data from the SQL Server database is available upon request. Raw data files are available via ProteomeXchange with identifier PXD055594.

## Supporting Information Available

Orbitrap X!TANDEM Supplemental gene symbols.xlsx (XLSX)
XTANDEM_ALB_peptides supplemental data (XLSX)
Supplementary Tables 1 and 2 and supplementary Figures 1–5. Effect of monoisotopic ion extraction on the counts of known plasma proteins (i.e., true positives) with mass tolerances; the impact of ion extraction of the peaks from heavy isotopes and hydrogen loss on plasma peptide counts (i.e., true positives) with the mass tolerances; the workflow of human plasma analysis by nano UPLC LC-ESI-MS/MS with a trihybrid axially harmonic orbital trap mass spectrometer; isotopic calculations for peptides across the mass distribution in this study using the ExPASy Isotopident tool; mass distributions of the Gaussian monoisotopic peaks from MS/MS spectra from peptide precursors matched to peptide sequences by X!TANDEM. The type I error cut off from the fit MS/MS spectra from Gaussian monoisotopic peaks and the Gaussian hydrogen loss at different mass tolerances and isotope monoisotopic peaks (PDF)

## Abbreviations

ACN: acetonitrile
ALB: albumin
BFPS: best fit per MS/MS spectra
BSA: bovine serum albumin
FDR: false discovery rate
LC-ESI-MS/MS: liquid chromatography-electrospray ionization-tandem mass spectrometry
LTQ: linear quadrupole ion trap
MH: [M + H]^+^, precursor mass
OIT: orbital ion trap
QA: quaternary amine (ammonium)
RCF: relative centrifugal force
SDS-PAGE: sodium dodecyl sulfate, polyacrylamide gel electrophoresis
SQL: structured query language
TRYP: fully tryptic peptide
