# Supplementary material for: Selected Ion Extraction of Peptides with Heavy Isotopes and Hydrogen Loss Reduces the Type II Error in Plasma Proteomics
Source: ACS Omega. 2025 Jan 2;10(1):281–93. doi: 10.1021/acsomega.4c05624 (PMC11739973; doi:10.1021/acsomega.4c05624)
Supplement: Supplementary file 3 — ao4c05624_si_003.pdf [file ao4c05624_si_003.pdf]

Selected ion extraction of peptides with heavy isotopes and hydrogen loss reduces the type II error in plasma proteomics

Jaimie Dufresne[1], Zhuo Zhen Chen[1], Pallvi Sehajpal[1], Peter Bowden[1], Ja-An Ho[2], Cheng-Chih Richard Hsu[2], John G Marshall[1,*]

[1]Department of Chemistry and Biology, Faculty of Science, Toronto Metropolitan University,

350 Victoria Street, Toronto, Ontario, Canada, M5B 2K3

[2] Department of Chemistry, National Taiwan University, Taipei 10617, Taiwan

*Corresponding author

SUPPLEMENTAL

Effect of monoisotopic ion extraction on the counts of known plasma proteins (i.e., true positives) with mass tolerances

The impact of ion extraction of the peaks from heavy isotopes and hydrogen loss on plasma peptide counts (i.e., true positives) with the mass tolerances

The workflow of human plasma analysis by nano UPLC LC-ESI-MS/MS with a trihybrid axially harmonic orbital trap mass spectrometer

Isotopic calculations for peptides across the mass distribution in this study using the ExPASy Isotopident tool

Mass distributions of the Gaussian monoisotopic peaks from MS/MS spectra from peptide precursors matched to peptide sequences by X!TANDEM

The type I error cut off from the fit MS/MS spectra from Gaussian monoisotopic peaks and the Gaussian hydrogen loss at different mass tolerances and isotope monoisotopic peaks

Supporting information are found in the files: Orbitrap X!TANDEM Supplemental gene symbols.xlsx and XTANDEM_ALB_peptides supplemental Data.xlsx.

Supplemental Table 1. Effect of monoisotopic ion extraction on the counts of known plasma proteins (i.e., true positives) with mass tolerances of ±0.01, ±0.05, ±0.1, ±0.2, ±0.3, or ±0.5 Da around the monoisotopic mass (delta mass, 0 Da). Peptides with similar observation frequencies in the blank noise recordings (493,541MS/MS) or random MS/MS spectra (30,000,000 MS/MS). A total of 524,867 MS/MS spectra from peptide precursors with 1,000 or more arbitrary detector counts were matched by X!TANDEM to 63,077 peptide sequences from a library containing 157,636 human protein accession entries. Type II error was computed with respect to the 7,698 peptides fit from MS/MS spectra by the X!TANDEM algorithm to known true positive plasma proteins based on their monoisotopic mass ±0.5 Da. The members of the Ig superfamily, were removed.

Gene Symbol	±0.01 Da	±0.05 Da	±0.1 Da	±0.2 Da	±0.3 Da	±0.5 Da
ALB	483	573	625	692	739	865
C3	333	389	400	419	431	447
FGA	252	271	278	291	300	315
CP	239	265	269	274	284	297
TF	219	247	268	281	288	311
APOA1	207	228	241	251	262	289
SERPINA1	204	230	242	246	255	273
APOA4	181	189	197	201	205	218
C4B_2	173	182	186	190	192	202
C4B	171	180	184	188	190	200
C4A	169	178	182	186	188	198
HP	150	169	175	189	196	215
FGB	146	177	181	189	191	199
ORM1	140	160	167	175	184	201
ITIH4	138	152	163	164	168	172
A2M	131	141	145	146	154	166
AZGP1	124	132	140	146	150	162
IGHG1	121	139	143	154	162	183
FGG	120	137	143	148	155	167
ORM2	113	120	127	131	139	152
HPX	107	112	112	118	122	129
GC	91	96	102	106	107	119
HPR	90	99	102	108	110	116
A1BG	88	96	97	100	102	112
SERPINC1	81	87	94	101	104	113
TTR	79	89	94	95	97	100
HBB	77	86	87	93	95	109
SERPINA3	76	86	87	91	91	98
ITIH2	74	86	89	95	99	103

CLU	71	74	75	77	81	84
SNC73	71	79	85	86	92	99
APCS	68	80	82	83	86	94
CFB	68	69	72	72	74	79
AAT	59	66	69	69	73	80
HBA2	59	63	63	63	67	70
CFH	58	65	68	72	74	79
HBA1	58	63	63	63	67	70
ITIH1	58	66	67	71	73	76
KNG1	53	70	73	74	74	80
AHSG	52	55	59	60	61	65
AFM	51	56	57	58	59	62
HBD	51	52	52	56	57	60
APOB	50	51	53	56	57	60
HRG	48	48	51	53	57	60
LRG1	46	54	55	56	57	60
APOE	45	48	48	49	50	51
C9	45	52	52	53	58	62
APOA2	43	48	51	56	62	65
SAA1	43	50	50	50	50	53
GSN	39	46	48	51	52	58
Sum	5713	6351	6613	6896	7141	7698
Type II error	35%	21%	16%	9%	7%	0

Supplemental Table 2. The impact of ion extraction of the peaks from heavy isotopes and hydrogen loss on plasma peptide counts (i.e., true positives) with the mass tolerances of ±0.1, ±0.2, ±0.3, or ±0.5 Da around the Gaussian peaks at −3, −2, −1, 0, +1, +2, +3, +4, and +5 Da. Peptides detected in blank noise recordings (493,541 MS/MS) or random MS/MS spectra (30,000,000 MS/MS) with similar observation frequencies, including the members of the Ig superfamily, were removed. A total of 524,867 MS/MS spectra from peptide precursors with 1,000 or more arbitrary detector counts were matched by X!TANDEM to 63,077 peptide sequences found in a library of 157,636 human protein accessions. Type II error was computed with respect to the 17,407 peptides fit by MS/MS spectra by the X!TANDEM algorithm to known true positive plasma proteins with Gaussian rearrangements and integer peak mass of ±0.1 Da. The type II error rate is computed with respect to all successful MS/MS fits from X!TANDEM. The members of the Ig superfamily, were removed.

**Gene Symbol**	**± 0.1 Da**	**± 0.2 Da**	**± 0.3 Da**	**± 0.5 Da**	**All MS/MS**
ALB	1617	1936	2192	2851	3007
C3	703	771	818	951	967
CP	533	560	581	649	652
FGA	498	548	580	648	664
APOA1	425	481	517	631	649
SERPINA1	422	477	525	614	624
ORM1	383	427	474	571	600
HP	371	448	475	574	596
FGB	367	402	422	478	484
C4B_2	351	372	391	454	457
APOA4	346	372	398	443	448
C4B	345	368	387	450	453
C4A	333	356	376	437	442
AZGP1	308	346	368	410	415
ITIH4	305	321	338	366	367
ORM2	286	310	356	411	419
A2M	255	279	310	361	364
FGG	237	273	293	348	349
HPX	217	244	268	311	322
A1BG	212	237	246	282	284
GC	206	227	241	276	283
SERPINC1	184	202	211	244	246
CFH	173	182	197	227	236
SERPINA3	172	190	196	219	219
HBB	163	192	209	260	262
HPR	154	182	192	227	233
ITIH2	136	152	161	181	188

ITIH1	134	151	162	182	185
APCS	132	142	153	173	179
CLU	131	143	153	169	172
AHSG	124	141	154	190	195
KNG1	123	137	142	162	167
HBA1	121	125	135	149	152
C1	118	138	149	179	185
HBA2	117	121	132	146	149
CFB	105	112	121	147	147
GSN	101	106	116	134	136
HBD	99	120	127	144	144
AFM	97	102	109	119	119
AAT	93	107	113	135	135
LRG1	88	93	97	107	107
SAA1	86	94	97	109	110
C9	84	88	101	120	120
APOA2	83	95	109	136	137
RBP4	78	84	94	121	121
HRG	77	88	93	111	111
APOB	76	86	91	110	111
PGLYRP2	75	85	93	115	116
APOE	73	78	82	86	86
AT3	66	75	81	90	93
Sum	11,983	13,366	14,426	17,008	17,407
Type II error	45%	30%	20%	2%	0%

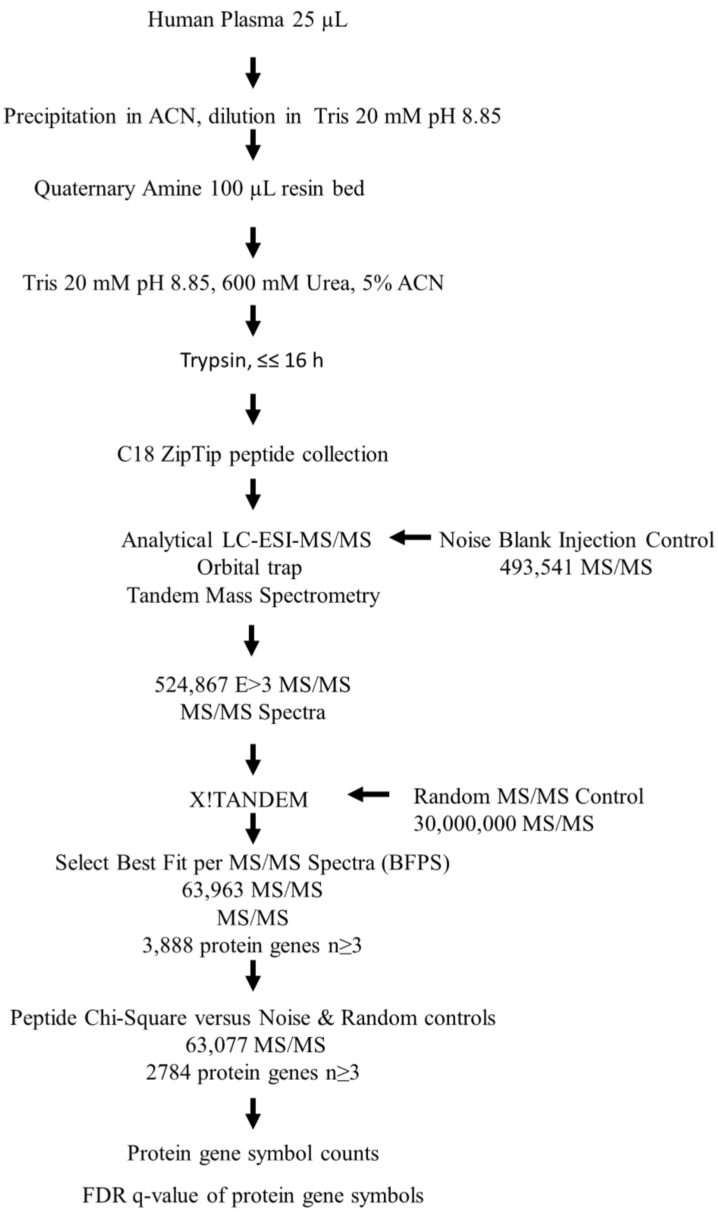

Supplemental Figure 1. The workflow of human plasma analysis by nano UPLC LC-ESI-MS/MS with a trihybrid axially harmonic orbital trap mass spectrometer leading to the identification of 3,888 protein gene symbols from a fit of 524,867 precursor (E>3) experimental MS/MS spectra to 63,077 MS/MS fully tryptic human peptides using the rigorous X!TANDEM algorithm. The peptides with observation similar to the analytical control of 29 blank noise injections (493,541 MS/MS) were removed by the SQL Server. The peptide counts from experimental samples were then compared to the statistical control of random fragmentation spectra (30,000,000 MS/MS) to compute the corrected observation frequencies of 2,784 proteins.

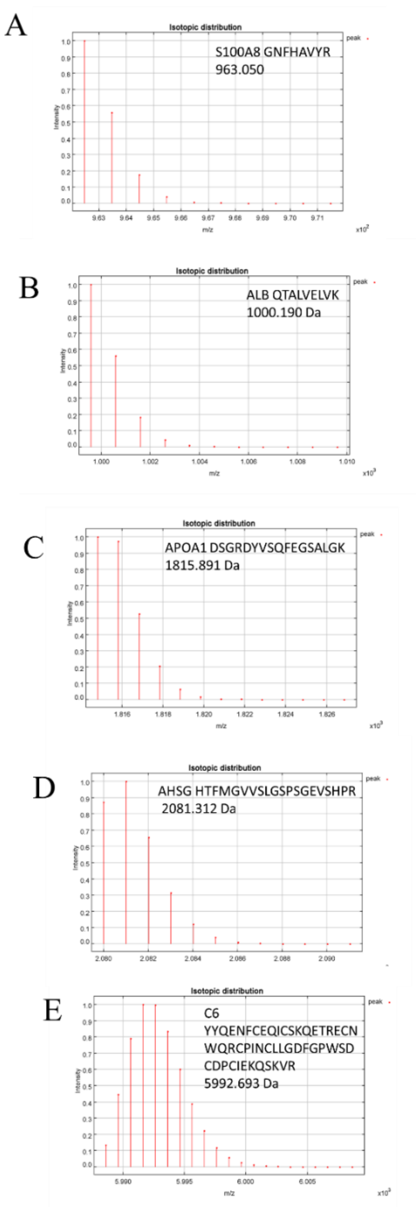

Supplemental Figure 2. Isotopic calculations for peptides across the mass distribution in this study using the ExPASy Isotopident tool. A, S100A8 peptide GNFHAVYR, 963.050 Da; B, ALB peptide QTALVELVK, 1000.190 Da; C, APOA1 peptide DSGRDYVSQFEGSALGK, 1815.891 Da; D, AHSG peptide HTFMGVVSLGSPSGEVSHPR, 2081.312 Da; E, C6 peptide YYQENFCEQICSKQETRECNWQRCPINCLLGDFGPWSDCDPCIEKQSKVR, 5992.693 Da.

MONO ISOTOPIC

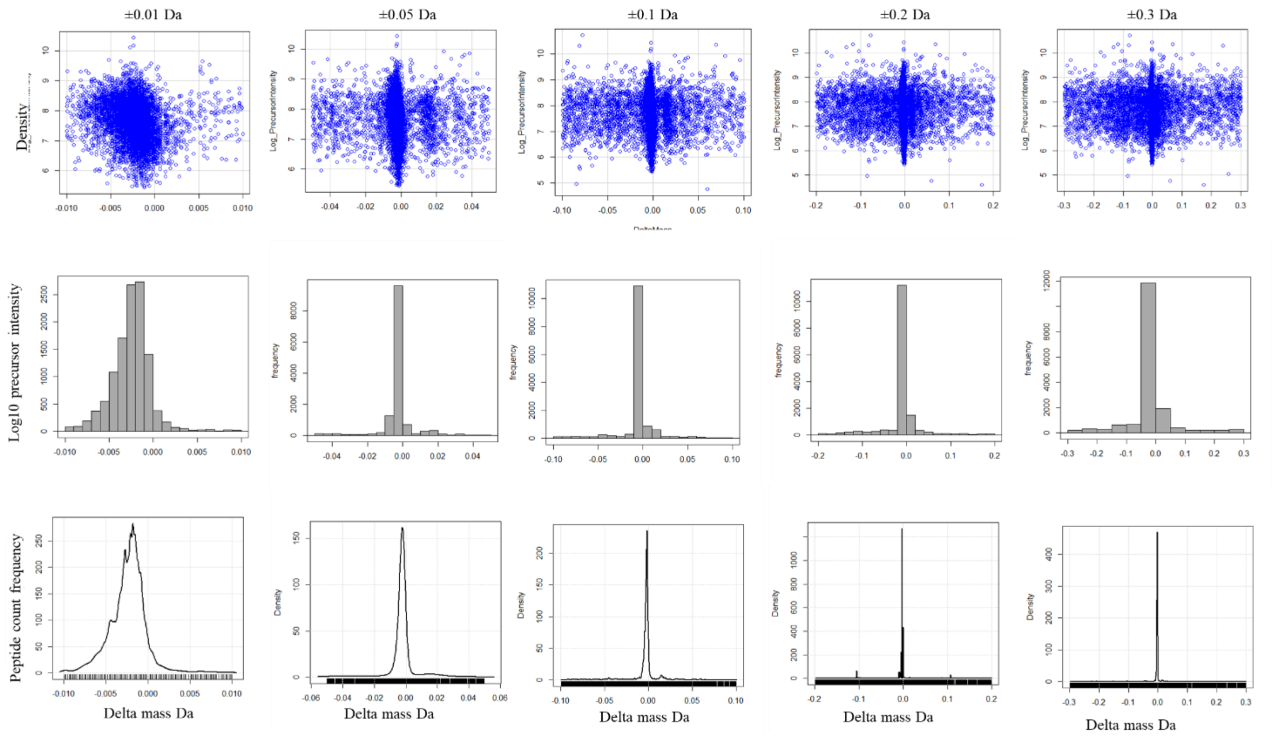

Supplementary Figure 3. Mass distributions of the Gaussian monoisotopic peaks from a total of 524,867 MS/MS spectra from peptide precursors with 1,000 or more arbitrary detector counts that were matched to 67,898 peptide sequences by X!TANDEM.

REARRANGEMENTS AND ISOTOPES

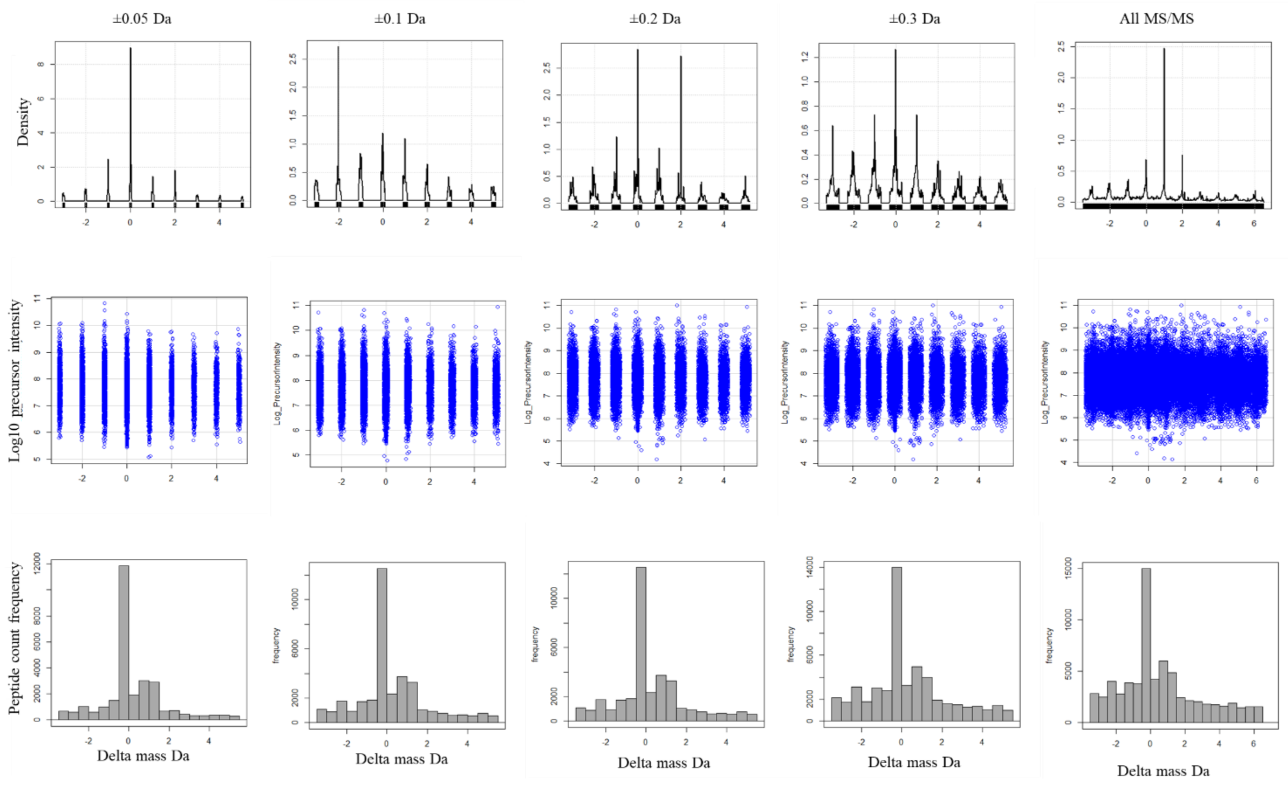

Supplementary Figure 4. Mass distributions of the Gaussian hydrogen loss and monoisotopic peaks from a total of 524,867 MS/MS spectra from peptide precursors with 1,000 or more arbitrary detector counts that were matched to 67,898 peptide sequences by X!TANDEM.

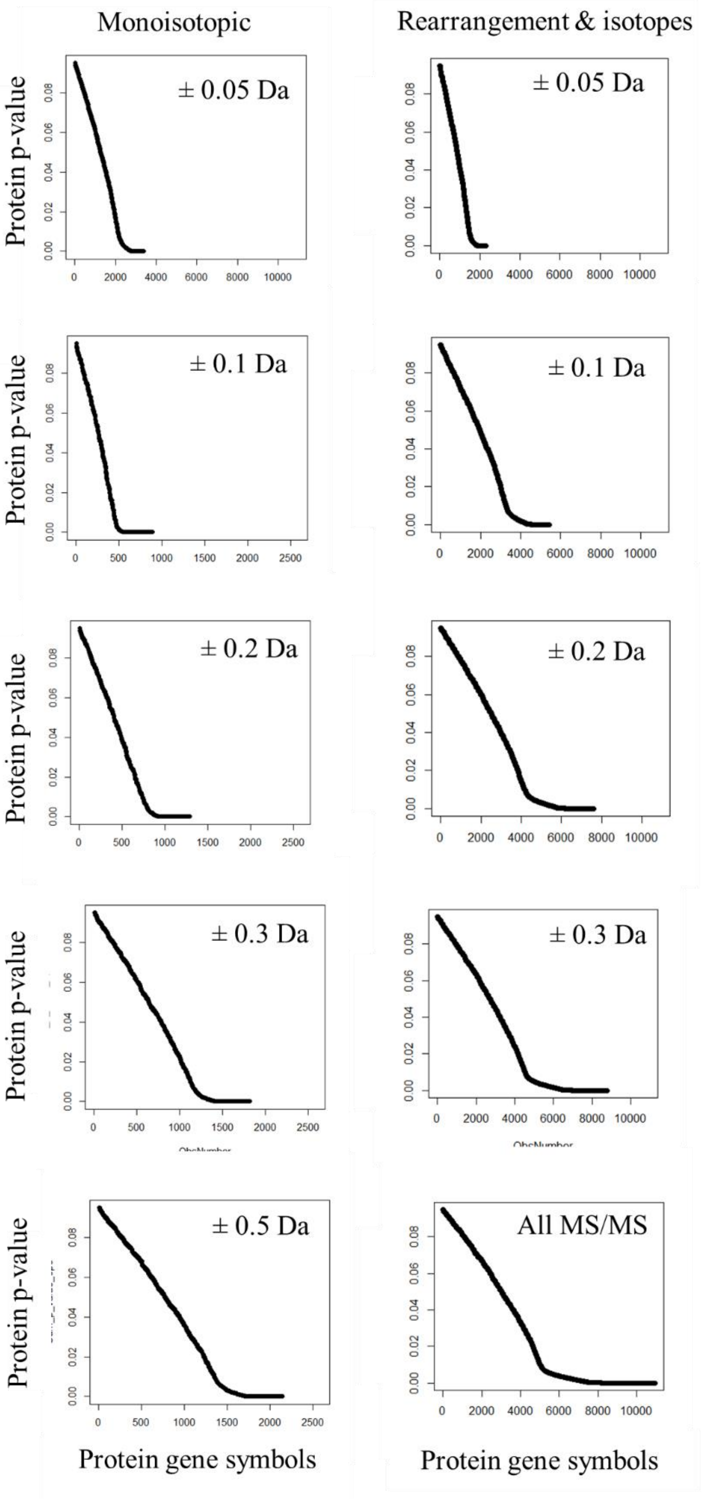

Supplementary Figure 5. The type I error cut off from the fit MS/MS spectra from Gaussian monoisotopic peaks and the Gaussian hydrogen loss at different mass tolerances and isotope monoisotopic peaks versus the fit of 524,867 MS/MS spectra from peptide precursors with 1,000 or more arbitrary detector counts that were matched to 67,898 peptide sequences by X!TANDEM.
